# Rarecare: A policy perspective on the burden of rare diseases on caregivers in Latin America

**DOI:** 10.3389/fpubh.2023.1127713

**Published:** 2023-03-02

**Authors:** Ariadne Guimarães Dias, Antoine Daher, Lucy Barrera Ortiz, Sonia Carreño-Moreno, Sylvia R. Hafez H, Angela Marie Jansen, Mariana Rico-Restrepo, Lorena Chaparro-Diaz

**Affiliations:** ^1^Institutional Relations, Casa Hunter, São Paulo, Brazil; ^2^Nursing Faculty, Universidad Nacional de Colombia, Bogotá, Colombia; ^3^The NOA Project, Panama, Panama; ^4^Americas Health Foundation, Washington, DC, United States; ^5^Americas Health Foundation, Bogotá, Colombia

**Keywords:** caregiver, rare disease caregiver, Latin America, caregiver burden, legislation supporting caregivers, policy, rare diseases

## Abstract

In Latin America (LA), 40–50 million people live with rare diseases (RDs) that require constant monitoring, care, and attention. Caregivers help them with their basic life activities and medication administration, which they would otherwise be unable to perform. Family caregivers complement healthcare and social security systems; however, their unpaid work is often underappreciated and under-protected. Recognizing the need to address these unrecognized and undervalued women, the Americas Health Foundation (AHF) convened a panel of LA experts on caregiving for people with RDs to provide recommendations to support the undervalued family caregivers. A panel of LA experts in caregiving for RDs were given questions to address the challenges faced by family caregivers of people with RDs in LA. During a 3-day conference, the panelists' responses were discussed and edited until the panel agreed on recommendations to address the challenges. The identified challenges for caregivers included physical, emotional, and economical areas. Caregivers, primarily women, experienced physical pain, and social isolation, and were forced to pay substantial out-of-pocket expenses in their caregiving roles. Brazil and Colombia are at the forefront of policies to protect caregivers and their experiences in attempting to provide for this group are outlined as case studies for what is possible in LA. Finally, recognizing that caregivers must be included in formulating, executing, and evaluating care policies for people living with RDs and that the caregivers themselves require social assurances, the panel suggested policy objectives aimed at protecting caregivers of people living with RDs. The recommendations ranged from recognizing the role of the family caregiver as an essential supplement to the formal healthcare system to providing financial assistance, training, and workplace protection, among others. Finally, monitoring and evaluating the impact of policies is necessary to ensure that LA is moving forward in caring for family caregivers for people with RDs.

## 1. Introduction

Approximately 40–50 million people live with a rare disease (RD) in Latin America (LA) (1, 2). RDs are chronic illnesses that require continuous monitoring, care, and attention. They impact not only the patient but also their family, friends, and community, who together face challenges and uncertainty innate to RDs (3). Caregivers support the basic activities and caretaking decisions of a person with disabilities, who, without this assistance, would not be able to perform them. This role can be exercised by a professional or a non-professional. However, parents or close relatives are usually the primary caregivers when a child is diagnosed with a RD. Thus, the number of informal or family caregivers is at least double that of patients, translating into over 80 million primary caregivers of people living with RDs in LA. Family caregivers are crucial support to some of the most vulnerable populations, including those with RDs. This group, disproportionately composed of women (4–10), provides care that complements healthcare and social security systems, but the value they contribute to economies and societies through their unpaid work is often underrecognized and under-protected.

The definition of RDs varies throughout LA. Some countries, like Bolivia, Ecuador, Paraguay, Peru, and Venezuela, do not have a specific definition for RDs. Meanwhile, Brazil considers RDs to be conditions affecting <65 individuals per 100,000, based on the World Health Organization's definition. Colombia defines RDs as conditions affecting fewer than 1 in 5,000 individuals. Argentina, Chile, Mexico, Panama, and Uruguay follow the European Union's definition, which considers RDs to be conditions affecting fewer than 1 in 2,000 individuals. The lack of a consistent definition across the region makes it difficult to accurately estimate the number of people affected by RDs, develop standard policies, integrate programs and registries, and allocate research funding (11).

Likewise, the policy landscape for RD throughout the region is heterogeneous. Several countries, including Argentina, Colombia, Ecuador, Mexico, Paraguay, and Perú have enacted national laws to address RDs (12). Brazil issued an Ordinance in 2014 but has not yet passed national legislation. In Chile, the Ricarte Soto Law was initially focused solely on RDs, but was later expanded to provide financial protection for high-cost diseases. Mexico's law is the least detailed and defines RDs based on incidence, while Peru and Argentina have comprehensive laws but are still in the process of implementation.

Colombia's law was the first established and Chile's is the most recent, with a clear difference in approach. Colombia's law is a social protection measure for RD patients, whereas Chile's law restricts the patients that are covered and provides financial protection some (13). Nevertheless, even the countries that have mature and well-structured RD policies have struggled with implementation. Further, while these policies address and protect the patient, they do not consider the caregiver.

The need to develop social policies to support the caregiver population is urgent. According to the December 2021 United Nation Resolution “Addressing the Challenges of People Living with a RD and their Families,” it is necessary to advance discussions on the main pillars of the Sustainable Development Goals, such as access to education and decent work, poverty reduction, addressing gender inequality, and promoting social participation (14).

Of note, caregivers must often assume the tasks of caring for their loved ones without the preparation required and/or without the support of health institutions. Depending on the care receiver's disease, caregivers carry out various activities. They assist with bathing, dressing, feeding, and grooming. They shop, cook, houseclean, and coordinate medical appointments. They also often perform health-related tasks, such as changing breathing tubes, administering medication, conducting physiotherapy, and feeding through gastrostomy tubes, for which they generally have received basic or no instruction (15–17).

These caregiving tasks are usually undertaken at personal responsibility and expense. Paradoxically, when care receivers are residents in nursing homes or patients in hospitals, paid health workers perform the aforementioned tasks. Informal caregivers, by contrast, carry out these essential services for no pay. Thus, caregivers comprise a substantial but largely unrecognized workforce throughout the region. In high-income countries, family caregivers provide more than 80% of the care required by individuals with long-term conditions, contributing billions of dollars of unpaid work per year to the healthcare system. This unpaid caregiving is likely amplified in LA, where healthcare systems are overburdened, and access to care is limited.

Limited and inconsistent support is available to caregivers for RD in LA, burdening the economy and negatively impacting the physical, emotional, and financial health of caregivers and care receivers. Policies are lacking in the region to ensure measures that recognize the vital role of informal caregivers in the health care and social security systems. Recognition would be possible through actions that address caregiver needs through community support, financial assistance, and workplace protections. This manuscript explores the landscape of caregivers for people living with RDs in LA, using Brazil and Colombia as case studies, given that these two countries are at the forefront of policies for RD in LA. Recommendations to advance policies that address caregivers' needs and rights in the region are provided.

## 2. Methods

The Americas Health Foundation (AHF) identified six experts in caregiving for people with RD from Brazil, Colombia, and Panama. They were convened for a 3-day virtual meeting on July 18–20, 2022, to develop recommendations for supporting caregivers of people with RD. AHF conducted a literature search using PubMed, MEDLINE, and EMBASE to identify nurses, advocates, and researchers from LA who have published in the field of caretaking and RD since 2017 to compose the panel. Augmenting this search, AHF contacted opinion leaders from LA to corroborate that the list of individuals adequately represented the necessary field. All the experts who attended the meeting are named authors of this manuscript. An AHF staff member moderated the discussion. The authors retain complete control over the content of the paper.

Search strategy: AHF conducted a literature search using PubMed, MEDLINE, and EMBASE for any publications on caregivers for people with RDs. The following search terms were used: “caregiving for people with rare diseases,” “caregiver policy,” “rare disease policy,” “burden of caregiving,” “economic impact,” and “caregivers” in combination with “Latin America,” “Brazil,” “Colombia,” and “Panama” from 01/01/2017 until 01/01/2022. The articles identified were in English, Portuguese, and Spanish. Particular attention was paid to identifying literature and research from LA.

AHF developed specific questions to address the challenges and needs of caregivers for people with RDs in LA as well as existing policy addressing the topic and assigned one to each panel member. A written response to each question was drafted by individual panel members based on the literature search and personal expertise. The entire panel reviewed and edited each narrative during the 3-day conference through numerous rounds of discussion until a total agreement on the content of the manuscript was reached. The recommendations developed were based on the evidence gathered, expert opinion, and personal experience and were approved by the entire panel. After the conference, the final manuscript was distributed by email to the panel for review and approval.

## 3. Results

### 3.1. Burden of caregiving

Being a caregiver for a person with a RD, even for a short period, can transversely and permanently impact the caregiver's life (18). Caregivers perform daily activities that require dedicating between 12 and 24 h to assist a person's partial or total dependency. According to the Corbin and Strauss Chronic Disease Trajectory Model, the burden felt by caregivers is understood as the relationship between the difficulties, dangers, and problems arising from the caregiver's experience (19). Another perspective by Jaime Cerda proposes an analysis of the financial implications of having a chronic disease and exercising its care for the caregiver-patient dyad, including the impact on work, income generation, and quality of life (QoL) (20). The burden of caregiving is heightened in LA, where uncertainty and limited medical, educational, social, and financial resources negatively impact caregivers' psychosocial and familial life (3, 21–24).

### 3.2. Characterization of caregivers of people with RDs in LA

Caregivers of people with RDs around the globe share many commonalities and face similar challenges. Several studies from Europe (22, 25, 26) and the US (27, 28) describe caregivers face challenges primarily related to interactions with the health system and emotional, mental, and physical strain. Commonly reported challenges include achieving a diagnosis, communication with medical professionals, bureaucracy and fragmentation in healthcare systems, and lack of information about the disease. Caregivers report feelings of loneliness, abandonment, grief, guilt, and stress. Negative impacts are also seen on relationships with partners or family members and social circles. Many caregivers deal with anxiety and depression and some face exhaustion and somatic manifestations. Additionally, the financial burden of RDs is present as a challenge across the board due to the high costs associated with most RDs and because caregivers are often forced to abandon their main source of economic income.

Research is necessary for LA to validate what is observed by those involved in the day-to-day care of people with RDs and advocate for public policies that defend the interests of this population. In response to this need, Casa Hunter, a Brazilian non-profit organization that aims to ensure public solutions and sensibility for RDs, conducted a national survey with the support of the Brazilian Federation of Rare Diseases Associations, Febrararas, with the participation of over 500 caregivers of people with RDs. While data on chronic disease caregivers in other LA countries exists, this large-scale survey study was unique in providing a glimpse into the lives of RD caregivers in the region.

In line with global and LA trends, women were found to be the primary caregivers (91%); typically, they were the patient's mother (81%), who spent most of her time with the patient (98%, 7 days a week). Most caregivers (62%) did not have formal employment, and almost half (46%) resigned from their paid work to dedicate themselves fully to caregiving. Seventy-two percent of women lived with the patient's father, and 78% received financial assistance from him. Sixty-five percent reported not feeling recognized fully for their work as caregivers. Daily tasks included accompanying the patient to medical appointments, school, and other activities (92%), purchasing, and preparing food (80%), and performing personal hygiene (73%).

These data concur with international research that found caregivers are predominantly women, often the mothers of people living with RDs, and are in young adulthood (4, 7, 29, 30). Additionally, a Colombian study reported that 83% of caregivers for Huntington's Disease were women, averaging 32 years old, of which 30% did not previously know about the disease (31). In Colombia, caregiver activities are concentrated in the eastern region (rural areas of Colombia) and lower in the central region (urban areas). The contribution of men to care increased as the socioeconomic level rose (32). According to a study conducted by the National Alliance of Caregiving (NAC) in the US, about 53 h per week are dedicated to caring for children living with RDs, compared to 30 h a week for general child caregiving (10).

### 3.3. Physical and mental health burden of RDs

Taxing demands of caregiving and insufficient community support often compromise the physical and mental health wellbeing of RD caregivers in LA. In the Casa Hunter survey, the primary complaints reported were bodily pain (79%), poor sleep quality (60%), and insufficient energy to complete daily activities (82%). Social interactions were also affected due to caregiving needs, with 72% reporting feeling lost and 68% feeling emotionally isolated. An evaluation of the effort index of family caregivers of patients with Huntington's disease in Colombia revealed a high-effort index of 39%, a half-effort index of 34.8%, and physical exertion with the highest score (31).

Resilience, a core component of caregiver skillsets, was highlighted in the study. Despite the difficult conditions caregivers in Brazil confront, respondents reported caring for the patient made them more conscious of their internal strengths (95%) and more self-confident (87%). Strong social networks (81%) and close friends and relatives (79%) were also reported as positive supports. Together, these data demonstrate countless ongoing opportunities for research and analysis into the QoL of caregivers and their families.Financial Burden of Caregiving.

In Brazil, finances revealed more of caregivers' challenges. Most (77%) reported additional expenses following the patient's diagnosis, and 65% indicated not having enough money to make it to the end of the month. Less than half (47%) estimated a household income of two minimum wages, or US$ 447.20^*^ (^*^exchange rate 18/07/22). Although these numbers indicate a precarious financial situation, a correlation between caregivers' economic status and degree of education was not detected; 42% reported completing higher education.

Most (75%) employed caregivers felt less willing to work at their jobs, and 63% missed at least 1 day per month due to patient care. Sixty-one percentage were dissatisfied with their work quality, and 97% were preoccupied with thinking about the patient during work. Financial stress added to the overabundance of daily activities led in most cases to a feeling of continuous pressure, particularly for those with additional employment.

In Colombia, the National Administrative Department of Statistics (DANE) conducted a survey in 2022 (32) that showed that women spend two more hours per day than men doing unpaid work. Unpaid work activities include care for the older individuals and chronically ill. The annual value of care and support for household members in 2021 was USD$ 9,067,950 (29.1% of the total unpaid work). Women dedicated over 5 million hours per year to care and support for household members (77.7% of the total), while men contributed 22.3%.

**Figure 1:**
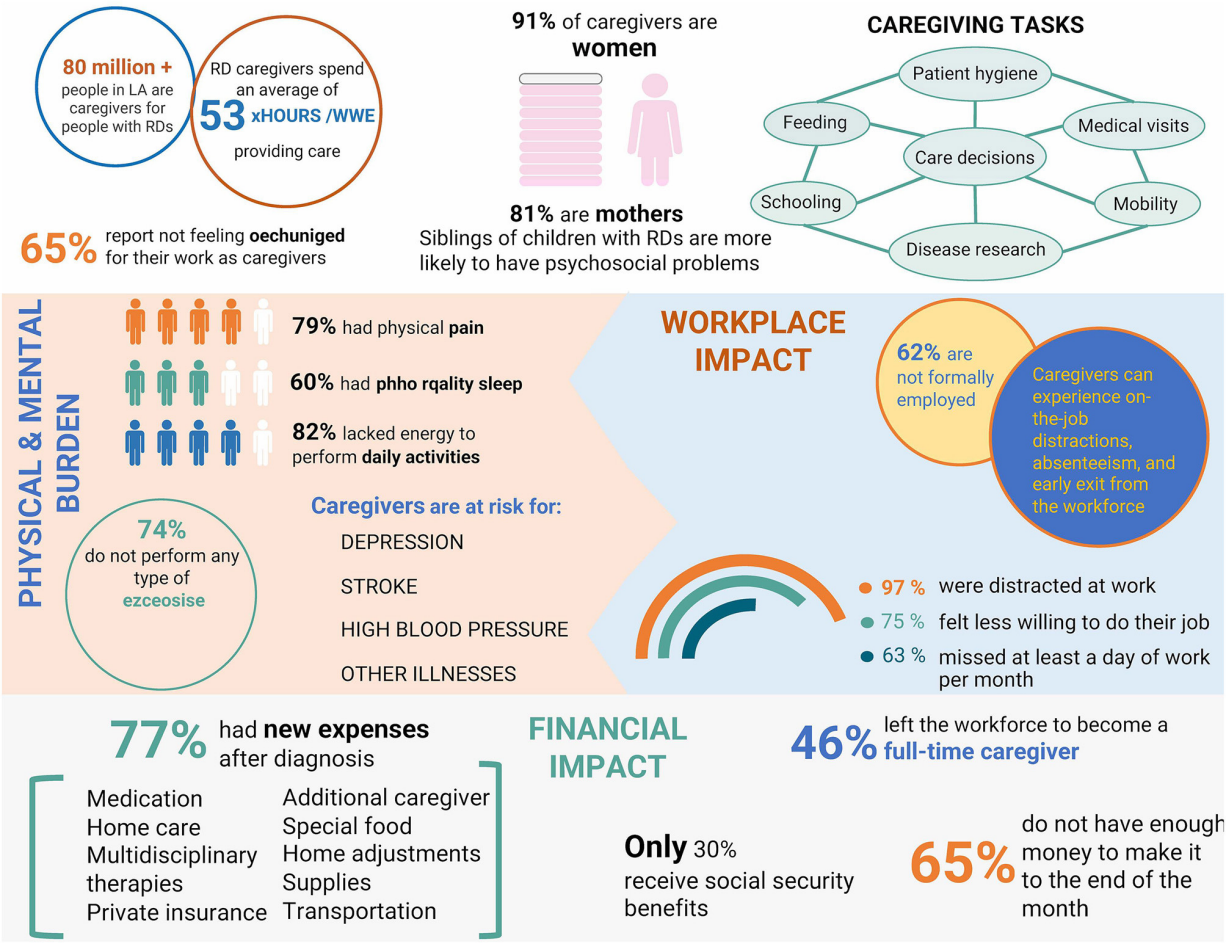
Schematic representation of the physical, mental, and financial burden on caregivers of people with rare diseases. LA, Latin America; RD, Rare diseases.

The prohibitive costs of RD are most attributable to lost income and increased healthcare expenditures. Ultimately, both are financially burdening to health systems, families, and caregivers. Caregivers sacrifice their careers or life projects and frequently incur lost income due to increased time requirements of patient care. They must also incur extra out-of-pocket expenses (33) given the increased demands for financial resources for transportation, public services, food, clothing, toiletries, home adjustments, medication, and supplies not covered by health insurance, as well as co-payments for healthcare services in some countries (34, 35). A schematic representation of the physical, mental, and financial burden on caregivers of people with RDs can be found in Figure 1.

### 3.4. Challenges and needs of caregivers for people with RDs

RD caregivers run at least two “marathons” in life: first, the search for a diagnosis (33) and second, access to treatment (when it exists), almost always marked by high cost—a synonym for orphan drugs worldwide. One of the greatest challenges caregivers of patients with RDs face relates to the insufficient knowledge about RDs in the medical community, requiring the caregiver and person with a RD to undergo a diagnostic odyssey before an appropriate diagnosis is obtained. LA reports are concordant with global data in that a lack of knowledge on RDs impacts the chance of early and accurate diagnosis (31, 36). These reports show that there is a need to increase physicians' awareness and education on the RDs (37–40). Most physicians were unable to differentiate RDs from commonly diagnosed disorders (37, 41). Unfortunately, medical students answering a poll in Poland over-estimated their knowledge of RDs (40). As expected, knowledge about RDs was highest among specialists and lowest among general practitioners (39), which poses a barrier as the first contact for most patients with RD is in primary care.

Because 95% of RDs do not have a cure, treatment and therapies are primarily used for palliative care and symptom management. Caregivers face challenges in meeting their patient care obligations due to difficulties in diagnosis, limitations or misinformation about treatment options, lack of scientific data about the condition, general expenses and out-of-pocket costs, lack of access to healthcare, lack of training to provide care, and social isolation (42, 43). Mastering the role of caregiver entails knowing how to access health information, navigating the healthcare system, managing symptoms, having the skills to make informed and shared decisions, developing behavior-oriented and problem-solving thinking, learning to manage technology in health support devices and knowing how to assist the patient in basic activities (44, 45). Even in today's connected world, most people in LA speak Spanish, Portuguese, or other native dialects, so language barriers hinder access to research and limit caregivers' ability to self-educate and seek the best care for their loved ones.

The systemic challenges to RD management in LA include comprehensive newborn screening programs, access to genetic and molecular testing, an insufficient number of geneticists and other specialists, and limited specialized care centers. Achieving quality care for RDs in LA requires better training, access, and national awareness; however, in the absence of progress, caregivers struggle to fill the need gap. On a personal level, caregivers' leisure time, independence, and ambitions are defaulted to patient care priorities leaving caregivers with little space to resolve guilt, frustration, anxiety, and depression (44). Overprotection, dependency, anxious attachment, and inadequacy characterize burdened relationships in caregiver-patient dyads. Ultimately, the systemic and personal challenges further fracture caregiver interpersonal, familial relationships, and social systems (45).

### 3.5. Global caregiver policy landscape

Countries worldwide have implemented integrated caregiver strategies to develop and coordinate the individual measures required to cover different needs. For example, the United Kingdom, Canada, and the US formally recognize caregivers' vital roles through national caregiver strategies. In the United Kingdom, the National Strategy for Carers was established in 1999, along with three Acts of Parliament: the Carers Act (Recognition and Services) (1995), the Carers and Disabled Children Act (2000) (46), and the Carers Act (Equal Opportunity) (2004) (8). The strategy was updated with reforms in June 2008 as part of a “New Deal for Carers” (47). The most recent action plan is outlined in Recognized, Valued, and Supported (48). This plan involves an integrated approach that engages six national government departments.

The Recognize, Assist, Support and Engage (RAISE) Family Caregivers Act became law in the US in 2018 (49). The RAISE Act requires the Department of Health and Human Services Secretary to develop a national caregiving agenda. This law includes finding ways to support family caregivers and strategies to improve federal programs that affect family caregivers. The law spurred the creation of a RAISE Family Caregiving Advisory Council charged with developing recommendations to drive future policies (5, 30, 32).

In Australia, the National Carer Strategy (2011) (50) supports the principles of the Carer Recognition Act (2010). It complements the National Disability Strategy, a 10-year plan for improving the lives of Australians with disabilities, their families, and caregivers. It seeks to value and respect caregivers and ensure they have rights, choices, opportunities, and capabilities to participate in economic, social, and community life.

### 3.6. Caregiver policy landscape in Brazil

In Brazil, there is little awareness of RDs, and the challenges and needs derived from them. As a result of advocacy efforts led by RD patient organizations in Brazil, the Ministry of Health passed Ordinance 199 of January 2014 establishing the National Policy for Comprehensive Care for People with RDs. Since then, the Legislative and Executive branches have focused on diagnosis, SUS (Brazil's national healthcare system) funding, and, when available, treatment access. However, the ordinance has not been fully implemented due to a lack of funding and enforcement.

In addition to the Federal Constitution (51) people with RDs and their caregivers usually depend on legislation designed for people with disabilities, documented since the 1960s. However, the RD community has specific needs that are not addressed by this law. Among the documents developed for the disabled, the Brazilian Law for the Inclusion of Persons with Disabilities (LBI) of July 2015 (52) is important to the RD community. Other laws include The Accessibility Act of December 2000 (53); the Complementation Program for Specialized Educational Assistance for People with Disabilities (54), and the Organic Law of Social Assistance (LOAS) (55). The former grants access to a monthly minimum wage to people with disabilities and the older individuals who, demonstrably, do not have the means to provide for their maintenance or to have it delivered by their families.

#### 3.6.1. Inclusion of persons with disabilities law

Comprising 127 articles, the Brazilian Law for the Inclusion of Persons with Disabilities (56) seeks to ensure and promote the exercise of fundamental rights and freedoms by people with disabilities, aimed at their social inclusion and citizenship. The law guarantees the disabled person's access to health, habilitation, and rehabilitation, but it does not address the effect of the disabled person's dependency on their caregiver. Although referenced in Chapter I, General Provisions, the caregiver is addressed peripherally throughout the document.

Among the small advances observed regarding protecting and training caregivers are mentions of the rights to (1) “Psychological care, including for their family members and formal caretakers” and (2) “Adequate and accessible information for people with disabilities and their family members about the health condition.” Chapter VII, “From the Right to Social Assistance,” recognizes the family as an environment of healthcare and ensures access to social assistance, healthcare, and financial stability for patients and caregivers. In this excerpt from the document, three articles seek social protection mechanisms in articles 39–41.

The document ends without any additional mentions of care for caregivers, although it is important to note that the National Registry of Inclusion of Persons with Disabilities (Cadastro-Inclusion) was created from the inclusion law with the objective of collecting, processing, systematizing, and disseminating georeferenced data that allows the identification and socioeconomic characterization of people with disabilities. This registry can be an important data source for understanding the percentage of disabled people due to RDs in Brazil and their economic situation and identifying caregivers.

**Figure 2:**
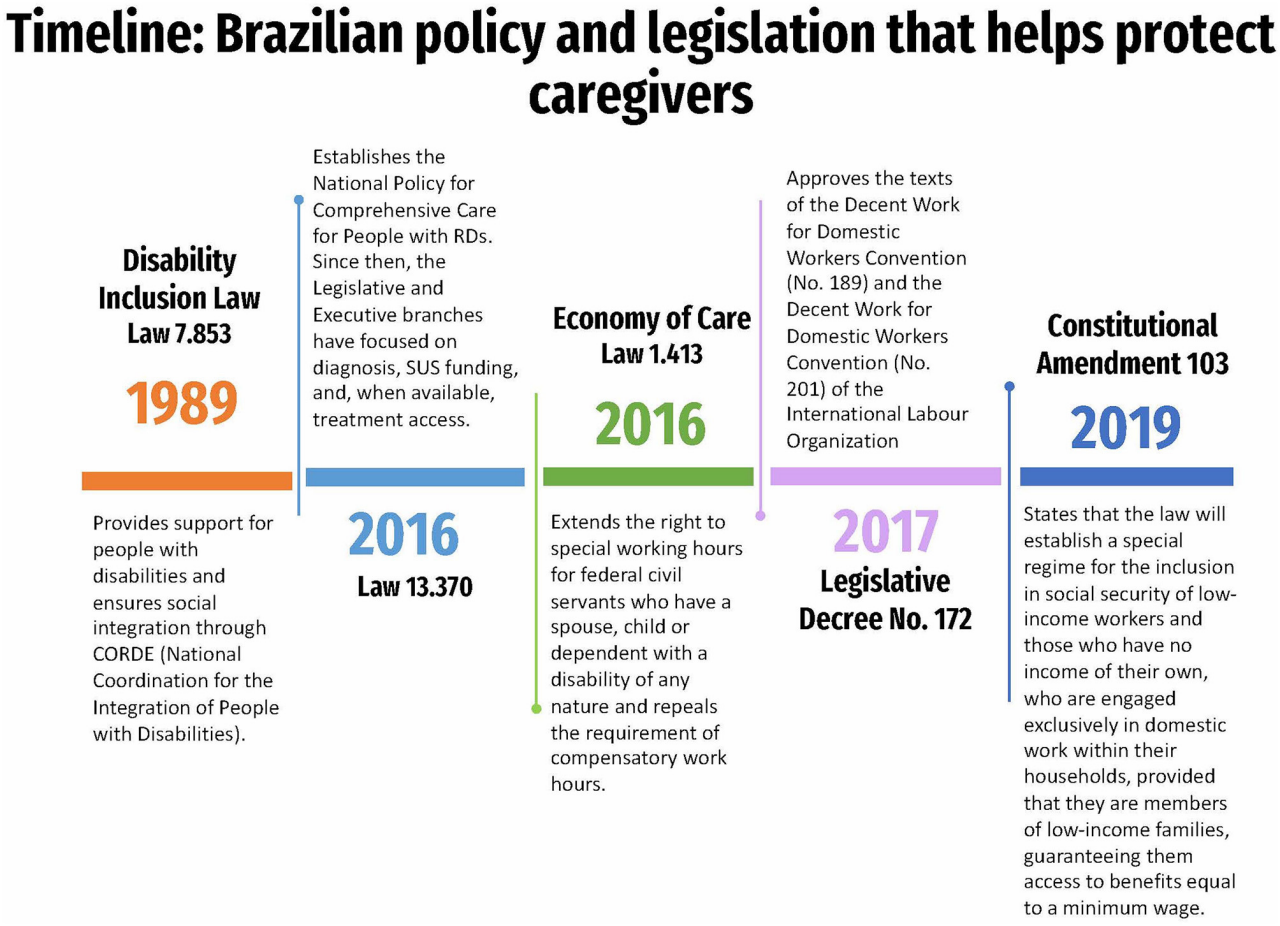
A timeline of the legislation passed in Brazil that helps protect caregivers. RDs, Rare disease; SUS, Sistema Único de Saúde (Brazilian public healthcare system).

In Brazil, a legislative project exists to provide taxpayers with the right to receive “caregiver assistance,” a benefit that must come from social security that provides resources to hire a caregiver or “personal assistant” service, whether an outside professional or a family member. However, this project has not yet been implemented, and its viability is threatened by the budgetary impact and the administrative logistics involved in granting the benefit (57). A timeline of the legislation in Brazil that helps protect caregivers can be found in Figure 2.

### 3.7. Caregiver policy landscape in Colombia

In Colombia, Law 1392 of 2010 (58) guarantees the protection of people living with RDs, defined as serious and neglected debilitating chronic diseases, and establishes the national RD registry to ensure access to care through the public healthcare system, which has improved the role of caregivers by covering needs specific to patient management.

The roles of family and informal caregivers are not recognized by Colombian legislation. Nevertheless, Article 46 of the Constitution (59) recognizes the family's responsibility to protect and assist people with dependencies. Essentially, the national constitution designates a social responsibility for caregiving to the family but does not determine the caregiver's rights. In some countries, there are economic subsidies for caregivers' services. This support is currently impossible under the existing healthcare system (Rulings T-154-14 and T-096/16 of the constitutional court) because caregiving responsibilities are delegated 100% to family members, who do not have any tributary or economic benefits when assuming this role, even under contexts of vulnerability.

Like Brazil, Colombia lacks laws protecting RD caregivers and has adopted the United Nations Convention on the Rights of Persons with Disabilities and Law 1618 of 2013, establishing the National Disability System. These are widely applied to people with RDs (23). In the framework of this policy, labor market inclusion and income generation of persons with disabilities are promoted, but little is said about caregivers. Also, this policy is not fully applicable to people living with RDs due to the often severely limiting nature of the diseases. A strategy promoted by this law is community-based rehabilitation, in which the Colombian Institute for Family Welfare (ICBF) and the National Learning Service (SENA) offer training and education.

The Statutory Law on Health (60) (Law 1751 of 2013) establishes the respect, protection, and compulsory nature of the right to health. It also dictates that the state must deliver special protection, and its institutions provide intersectoral and interdisciplinary attention to guarantee better conditions in care.

Law 1413 of 2010 (61) is one of the most significant advances in Colombian legislation. This law included the “economy of care,” defined as reproductive or domestic labor, which must register an economic contribution in the System of National Accounts, which measures women's contribution to economic and social development and has contributed to the formation of public policies and feminist movements. These measures can make it possible to recognize, reduce, and redistribute the overload of unpaid work within households for healthcare and care for children, the older individuals, and people with disabilities and serious illnesses. While these rules were significant legislative achievements, the second half of the equation, a system to effectively ease the burden of caregiving, is not being implemented. The National Development Plan for 2018–2022 was a first step toward establishing a National Care System in Colombia. However, it is still under design.

In Colombia, there have been at least four legislative initiatives for the recognition of family caregivers. Some have a market-based profile, while others are state-centered. The National Caregivers Policy bills of 2009 (62) and 2020 (63) resulted in a unified bill presented to congress in 2022 that was approved in a secondary debate (64). Unfortunately, these recent attempts are subject to funding availability, depend on the governments in power, and are not always a political and social priority. In that sense, the National Caregivers Policy under development has been criticized because of the lack of funding.

**Figure 3:**
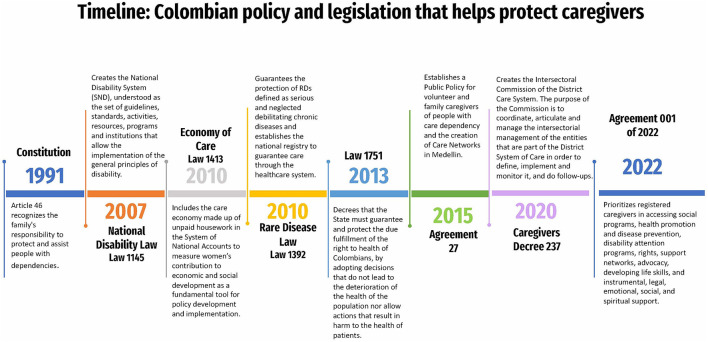
A timeline of the legislation passed in Colombia that helps protect caregivers. RDs, Rare diseases; LA, Latin America; RD, Rare diseases.

The evolution and integration of the legislative process aim to recognize, among others, unpaid personal care, or assistance of persons with disabilities or elders and unremunerated care of persons with permanent disabilities provided by relatives or another person. The constitutional principle guiding these projects has been human dignity, which has given rise to a substantial body of jurisprudence concerning the recognition of caregivers' freedoms and integrity, both physical and spiritual, as well as solidarity, because family members, by choice or obligation, assume the role of caregiver. A timeline of the legislation in Colombia that helps protect caregivers can be found in Figure 3.

Several Colombian cities have created local caregiver policies as part of initiatives focused on people with disabilities and the care of the older individuals. These policies entail strategies for improving the visibility of caregiving (such as the 3R's: Recognize, Redistribute, and Reduce), the empowerment of women, and prioritizing training and social benefits.

## 4. Conclusion

Creating visibility for caregivers of people living with RDs requires recognizing the value they, as a workforce, provide to economies, healthcare systems, and society. Despite these critical contributions, there is little formal recognition of this group and its needs in three key areas: lack of support programs to relieve the caregiving burden, limited or no financial assistance to ameliorate caregiving costs, and workplace accommodations to alleviate time and financial strain. Additionally, women's disproportionate burden of caregiving must be acknowledged as a factor worsening gender inequality.

Research into the burden and situation of RD caregivers in LA is needed to generate data that can be leveraged to create awareness and adopt strategies, political action plans, and laws that contribute to the wellbeing of RD caregivers and their families. In Brazil, these meetings have sparked interest from parliamentarians and public bodies. While small steps in policy advancements and efforts have been made in Colombia and Brazil, much work must still be done. Replicating and expanding local efforts that provide benefits to caregivers into national caregiver policies requires articulating sectors, one of the greatest challenges of any local or national policy. It is not only about the sum of services but having basic principles in the supply of care systems, monitoring, and evaluating implemented policies, and updating regulations. Recognizing, redistributing, reducing, redefining, and even rebuilding unpaid care are shared responsibilities for which LA has a historical debt to the caregivers of people living with RDs (65).

Undoubtedly, the path ahead is difficult and fraught with structural problems, ranging from seeking resources in the public system to developing the required infrastructure to support caregivers across LA. Only continuous dialogue efforts with national and international stakeholders and adopting good practices can accelerate this change—a need for LA and the world. “Alone, we can go fast, but together we can go further.” This should be a motto for inclusion in the RD world. The gap in LA RD caregiver policies must be bridged to lessen caregivers' burden, as they play a vital role in understanding the diseases, educating, caring for the patient, improving QoL, advocating for their charges, and ultimately finding treatment.

### 4.1. Recommendations

In formulating public policies, the continuous call to understand the legal, institutional, political, social, economic, and cultural context in which RDs are lived is reiterated to develop proposals aligned with reality. Caregivers must be included in formulating, executing, and evaluating care policies for people living with RD, together with patients, health professionals, providers, and public institutional representatives (66). A gender-guided approach is necessary, addressing women's disproportionate contribution to the economy of care through paid and unpaid work and combating a deepening in gender inequality due to the situation of vulnerability that caregiving creates. Several priority areas stood out across the policy and a recommendations review was conducted to develop recommendations tailored to LA (48, 50, 67). Among these are recognition, value, and respect for caregivers, access to education and training, financial security and workplace protection, benefits and support services, and improved health and wellbeing. The global policy precedents make clear that there is no one-size-fits-all approach, and each country must adopt best practices and recommendations to its local context and needs. This manuscript provides recommendations for the objectives that policies aimed at caregivers for RDs in LA should strive to meet (see Box 1).

Box 1 **Recommended objectives for policies aimed at caregivers of people living with RDs in LA (50, 67).**

1) Recognize RD caregivers as partners of formal healthcare and social service providers and include their voice in the planning, delivery and evaluation of the policies and programs that affect their lives
2) Implement a system to identify family caregivers through which the caregiver and care-receiver can be registered along with relevant information to access social programs and benefits
3) Determine the fiscal impact of RDs and caregiving for these disease on the economy, caregiver, and healthcare system
4) Foster mental and physical health of caregivers through access to psychosocial support
5) Facilitate access to coordinated, humane, comprehensive, and culturally appropriate healthcare
6) Ensure access to comprehensive training and education to promote optimal caregiving conditions that prevent risk for the caregiver and care-receiver
7) Encourage financial security, labor insertion, and workplace protection for caregivers
8) Provide financial assistance and social, tax, and/or housing benefits to ameliorate the costs of care, especially to those in vulnerable situations
9) Implement services and support to recognize, reduce, and redistribute caregiving burden at the community level
10) Leverage information and communication technologies to improve the care experience, lighten the load and improve the QoL of those involved
11) Encourage and support the creation and sustainability of caregiver and patient organizations for RDs
12) Monitor and evaluate the impact of implemented policies continuously and update regulations accordingly

## Author contributions

AGD, LB, SH, and LC-D: writing-original draft, investigation, formal analysis, and validation. AD: investigation, formal analysis, and validation. SC-M: writing-original draft. AJ: editing and critical revision for intellectual content and project administration. MR-R: writing-review and editing, visualization, conceptualization, methodology, and project administration. All authors agree to be accountable for the content of the work. All authors contributed to the article and approved the submitted version.
